# Ganglioneuroblastoma in a newborn with multiple metastases: a case report

**DOI:** 10.1186/s13256-017-1397-x

**Published:** 2017-08-29

**Authors:** Eva Gauchan, Prakash Sharma, Dilasma Ghartimagar, Arnab Ghosh

**Affiliations:** 1 Department of Pediatrics, Manipal College of Medical Sciences, Pokhara, Nepal; 2 Department of Radiology and Imaging, Manipal College of Medical Sciences, Pokhara, Nepal; 3 Department of Pathology, Manipal College of Medical Sciences, Pokhara, Nepal; 4 Manipal College of Medical Sciences, Manipal Teaching Hospital, Fulbari, Pokhara, 33701 Nepal

**Keywords:** Blueberry muffin rash, Case report, Ganglioneuroblastoma, Metastases, Newborn

## Abstract

**Background:**

Ganglioneuroblastoma is a tumor of peripheral neuroblastic tissue which occurs predominantly in the pediatric age group; it is a rare occurrence in the newborn period with only one case reported at birth to date.

**Case presentation:**

We report the case of a newborn male baby of Brahmin ethnicity from Nepal who presented with respiratory distress and blueberry muffin skin lesions after birth. A computed tomography scan showed a mass lesion in the posterior mediastinum, which was diagnosed as ganglioneuroblastoma on fine-needle aspiration cytology. He also had metastases to multiple sites including heart, lungs, skin and brain.

**Conclusions:**

Ganglioneuroblastoma is a rare tumor in newborns. Any newborn presenting with respiratory distress associated with blueberry muffin skin lesions should be evaluated for neuroblastic tumor.

## Background

Neuroblastic tumors fall third in line among the childhood cancers following leukemia and brain tumors. With an annual incidence of 7.6 per 1,000,000 population according to the Surveillance, Epidemiology and End Results (SEER) Registry, they are the most common solid extracranial tumors in children [1]. Representing the two extremes of neuroblastic tumors are neuroblastomas (most malignant) and ganglioneuromas (most benign). In between lie ganglioneuroblastomas (GNBs) – histologically containing a combination of primitive neuroblasts along with mature ganglion cells and hence considered malignant or potentially malignant. GNBs can further be subclassified into two types, that is, nodular and intermixed. It occurs with equal frequency in both sexes and most cases present before 10 years of age, with only one case at birth reported by Adam *et al*. [2].

## Case presentation

**Fig. 1:**
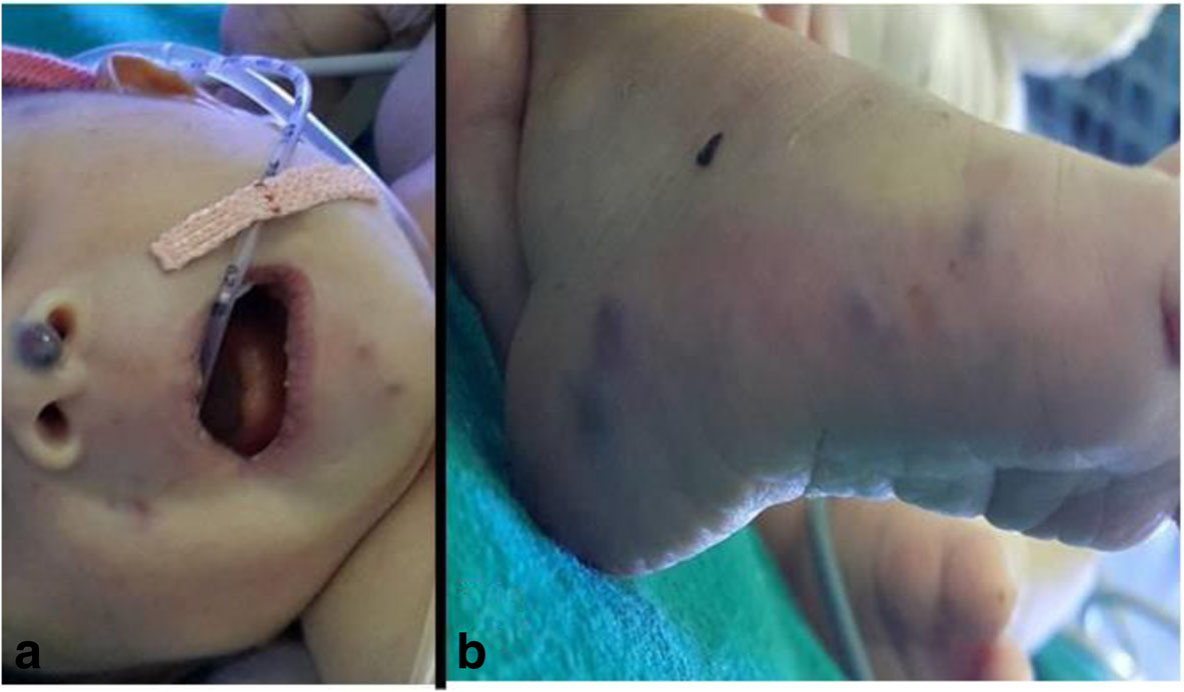
Blueberry muffin lesion over nose (**a**) and blueberry skin lesion over foot (**b**)

**Fig. 2:**
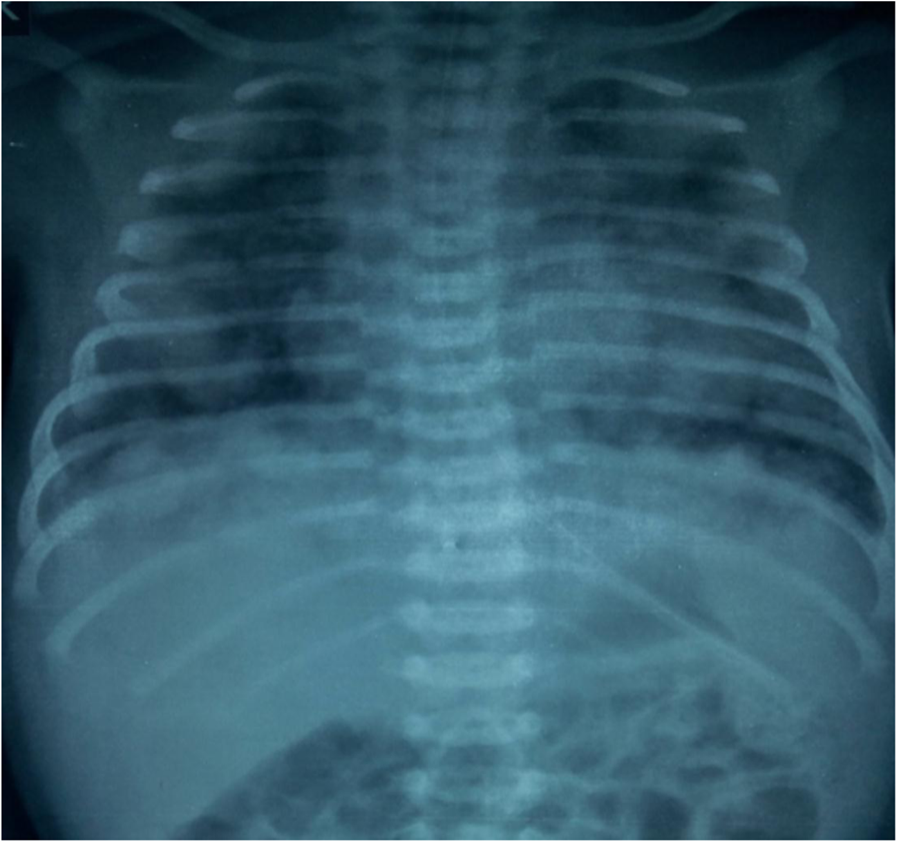
A chest X-ray, anteroposterior view, shows multiple nodules of varying sizes diffusely scattered in bilateral lung fields

**Fig. 3:**
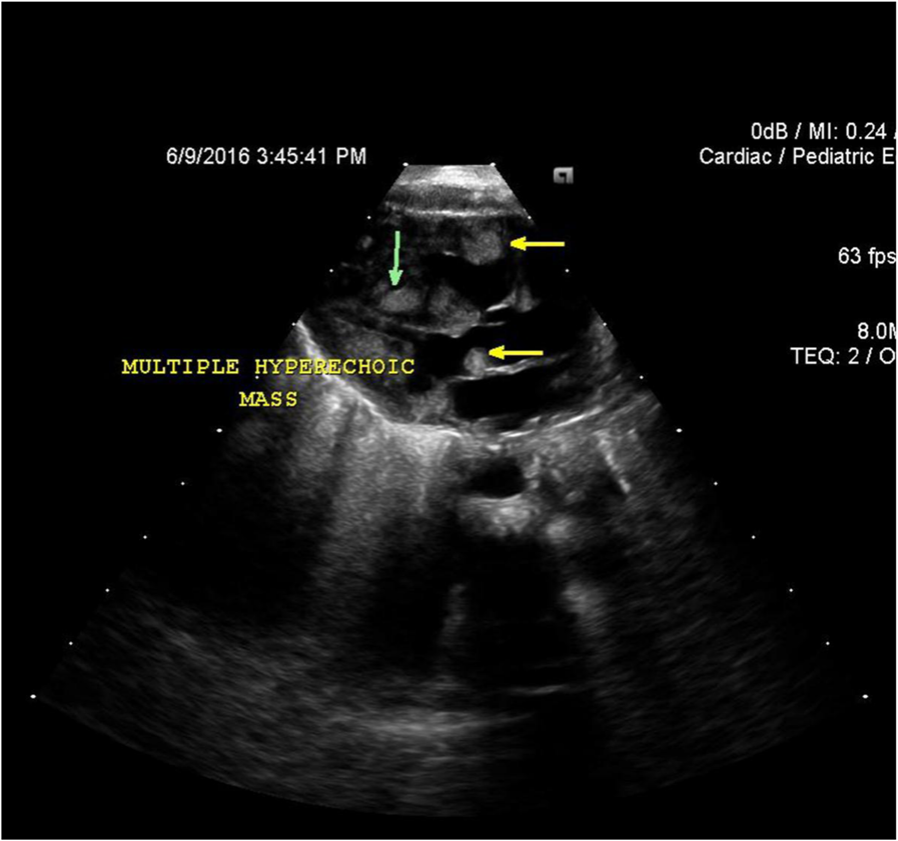
Echocardiogram showing multiple metastatic lesions in the right ventricular free wall (*upper yellow arrow*), aortic valve (*lower yellow arrow*) and right ventricular septum (*green arrow*)

**Fig. 4:**
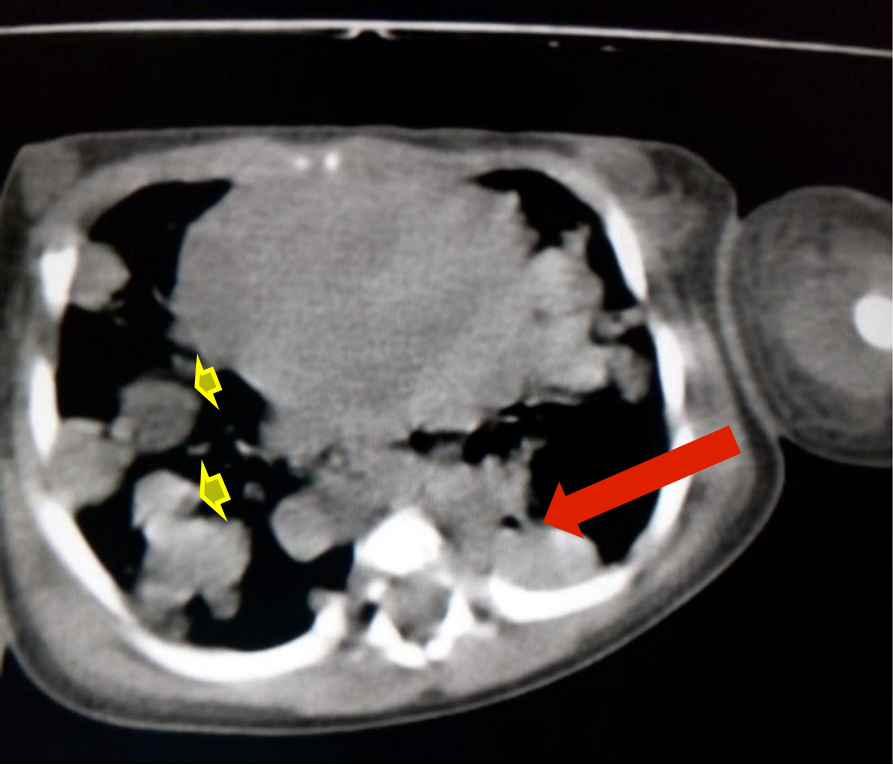
Non contrast computed tomography scan showing soft tissue density lesion in left paravertebral area (*red arrow*) along with multiple lung metastases (*yellow arrowheads*)

**Fig. 5:**
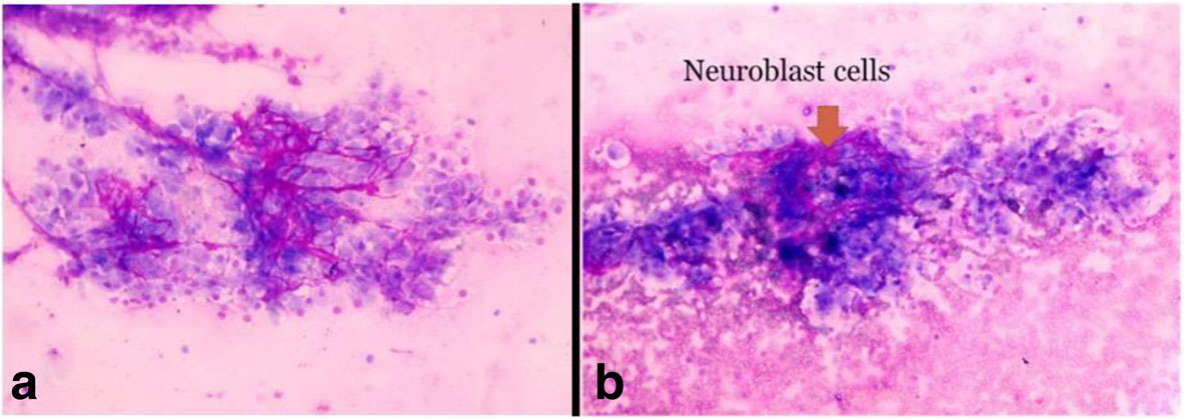
Ganglion cells in a papillaroid pattern with fibrillary stroma (**a**) and ganglion cells intermixed with neuroblasts (**b**). (Giemsa stain ×400)

A newborn baby of Brahmin ethnicity from Nepal presented within a few hours of life with respiratory distress and bluish skin nodules (blueberry muffin rash) all over his body. The baby was a male, weighing 2200 grams (below the tenth percentile for gestation), born at term to a 22-year-old primipara out of a nonconsanguineous union without any antenatal checkups. The baby was born with good Apgar scores and had multiple bluish skin nodules all over his body. Within 3 hours of birth, the baby developed severe respiratory distress and difficulty in feeding, with a temperature of 100.2° F. A physical examination showed a grunting, pale baby with peripheral cyanosis and tachypnea. There were multiple (23 in number) bluish nodules over his whole body; the largest one, measuring 1 × 1 cm, was over his nose (Fig. 1a, b). Auscultation of his chest revealed bilateral crackles and his cardiovascular system revealed tachycardia without murmurs. Abdominal and central nervous system examination findings were unremarkable. A chest X-ray showed fluffy opacities in the bilateral lung fields (Fig. 2) and an echocardiogram showed multiple well-circumscribed masses in the interventricular septum and left ventricular free wall (Fig. 3). An ultrasound of his abdomen was unremarkable except for the presence of simple cysts in his liver and kidneys. A computed tomography (CT) scan of his whole body was done, which showed a homogenously enhancing paravertebral soft tissue density lesion on his left side at D7-D9 level with metastatic lesions in his heart and lungs (Fig. 4), along with multiple supra and infratentorial petechial bleeds with perifocal edema in his brain. A TORCH panel of tests, done to rule out intrauterine infections, urinary vanillylmandelic acid (VMA) test and bone marrow examination results were normal. A CT-guided fine-needle aspiration cytology (FNAC) was performed and the smears showed several clusters and singly scattered ganglion cells. Some of the cells were seen in a papillaroid pattern with fibrillary stroma. The ganglion cells had abundant coarsely granular cytoplasm, round vesicular nucleus with centrally placed large prominent single nucleolus (Fig. 5a). Focal areas showed monolayer sheets of cells intermixed with neuroblast cells, the cells having moderate cytoplasm with round to oval nucleus with inconspicuous nucleolus (Fig. 5b). FNAC diagnosis was given as ganglioneuroblastoma, but as a biopsy could not be performed, we could not subtype it further. The parents were counseled about the diagnosis and available treatment options, but the baby was taken home against medical advice on 12^th^ day of life. On follow-up, it was found that the baby had died the day after discharge from hospital.

## Discussion

Respiratory distress is a very common problem in newborns. It can be due to several causes, notably pneumonia, sepsis, heart disease, congenital anomalies of the respiratory tract, and so on. GNB is a rare tumor in the newborn period. Rarer still is GNB presenting with respiratory distress. Most cases present before 10 years of age. After a literature search, we could find only one case which had presented at birth and another case at 13 days of life [2]. GNB arises from the sympathetic chain; anywhere from the adrenal medulla to the paravertebral sympathetic ganglia. The most common sites of origin are adrenal medulla (35%), extra-adrenal retroperitoneum (30–35%) and posterior mediastinum (20%) [3]. In 1981, Adam *et al*. reported a series of 80 cases all of which arose from the posterior mediastinum [2]. Rarely, they can be found in other sites, for example, neck, pelvis, lungs, nervous system and so on [3–8]. The mode of presentation depends on the area of involvement; they may present with mass effect or with complications of metastasis. Other features like malaise, irritability, weight loss, breathing difficulties, Horner syndrome, opsoclonus-myoclonus syndrome and peripheral neurologic signs can be seen. In our case, the baby had respiratory distress with tumor involving the posterior mediastinum. Two-thirds of cases present with metastatic bone disease, which manifests as limping and irritability (Hutchinson syndrome). After bone, the next common sites for metastases are bone marrow (40–60%), liver, and skin. In our case, the bone marrow aspirate was negative for evidence of metastasis and a liver scan did not show signs of disease. His skin, however, showed multiple blueberry muffin lesions. Lung and brain metastasis are said to be rare; but in our case there were soft tissue densities in the heart and lungs along with petechial hemorrhages in the brain, which are all very unusual sites for metastasis. In approximately 1% cases they present with metastasis with no discoverable primary tumor. In almost half the cases, the tumor is diagnosed incidentally (incidentaloma).

A CT scan is the standard modality of imaging to evaluate neuroblastic tumors [3]. It shows the origin and extent of tumor, vascular encasement, adenopathy, and calcifications. It also shows the extent of metastasis in the various organs. In the lungs, metastasis may be seen as discrete nodules or areas of consolidation, and brain metastasis, although rare, can be seen as hemorrhage, solid or cystic lesions [3]. Magnetic resonance imaging (MRI) is preferred over CT for evaluating intraspinal extension of primary tumor and for detection of hepatic metastasis in infants. Metaiodobenzylguanidine (MIBG) scintigraphy is another test that can be done to look for primary tumor and metastatic surveillance; however, its use in routine patient assessment is controversial as it does not separate cortical from marrow disease, which is important for staging of the disease. Similarly, technetium-99m medronic acid (MDP) scintigraphy can be done in all patients as part of initial screening to assess the metastatic burden [3].

Diagnosis is confirmed by histological study, preferably done by biopsy, which shows the typical small, round, blue cells with hyperchromatic nuclei, scanty cytoplasm, and ganglion cells characterized by abundant basophilic cytoplasm containing Nissl material, a large vesicular nucleus, and a prominent nucleolus. We diagnosed our case on the basis of FNAC. However, we could not differentiate the subtype of GNB as the baby was taken home before a biopsy could be performed. Bone marrow aspirate may show evidence of metastatic disease. However, Adam et al, in their study found negative marrow findings in 38 of 40 cases studied [2]. As in other catecholamine-producing neuroblastic tumors, elevated levels of VMA are found in approximately 65–90% of cases [3, 5].

Staging of disease is done as per the International Neuroblastoma Staging Study (INSS) guidelines based on clinical, radiological, and surgical features. Treatment depends on the stage and includes surgical excision, chemotherapy, radiotherapy, and bone marrow transplantation. Prognosis depends on the age of the patient and stage of disease. Age less than 1 year, primaries in the mediastinum, and stage 1–2 and 4S are usually associated with a better prognosis. However, in our case even though the primary site was in the mediastinum, presence of widespread metastases to the vital organs could have led to early mortality.

## Conclusions

Ganglioneuroblastoma is a very rare cause of respiratory distress in newborns. However, when respiratory distress is seen with blueberry muffin skin rashes, neuroblastic tumors should be ruled out. CT scan with CT-guided FNAC was found to be a good modality of diagnosis in our set-up.

## Abbreviations

CT: Computed tomography
FNAC: Fine-needle aspiration cytology
GBN: ganglioneuroblastoma
INSS: International neuroblastoma staging system
MDP: Medronic acid
MIBG: Metaiodobenzylguanidine
MRI: Magnetic resonance imaging
SEER: Surveillance, Epidemiology and End Results Registry
VMA: Vanillylmandelic acid
